# Evaluating the transduction efficiency of systemically delivered AAV vectors in the rat nervous system

**DOI:** 10.3389/fnins.2023.1001007

**Published:** 2023-01-23

**Authors:** Olivia J. Yang, Gabriella L. Robilotto, Firoj Alom, Karla Alemán, Karthik Devulapally, Abigail Morris, Aaron D. Mickle

**Affiliations:** ^1^Department of Physiological Sciences, College of Veterinary Medicine, University of Florida, Gainesville, FL, United States; ^2^Department of Veterinary and Animal Sciences, University of Rajshahi, Rajshahi, Bangladesh; ^3^Department of Neuroscience, College of Medicine, University of Florida, Gainesville, FL, United States; ^4^J. Crayton Pruitt Family Department of Biomedical Engineering, College of Engineering, University of Florida, Gainesville, FL, United States

**Keywords:** autonomic nervous system (ANS), sensory nervous system, dorsal root ganglion (DRG), peripheral nervous system (PNS), AAV9, AAV-retro, PHP.S, Adeno Associated Virus (AAV)

## Abstract

Gene delivery or manipulation with viral vectors is a frequently used tool in basic neuroscience studies. Adeno-associated viruses (AAV) are the most widely used vectors due to their relative safety and long-term efficacy without causing overt immunological complications. Many AAV serotypes have been discovered and engineered that preferentially transduce different populations of neurons. However, efficient targeting of peripheral neurons remains challenging for many researchers, and evaluation of peripheral neuron transduction with AAVs in rats is limited. Here, we aimed to test the efficiency of systemic AAVs to transduce peripheral neurons in rats. We administered AAV9-tdTomato, AAV-PHP.S-tdTomato, or AAV-retro-GFP systemically to neonatal rats *via* intraperitoneal injection. After 5 weeks, we evaluated expression patterns in peripheral sensory, motor, and autonomic neurons. No significant difference between the serotypes in the transduction of sensory neurons was noted, and all serotypes were more efficient in transducing NF200 + neurons compared to smaller CGRP + neurons. AAV-retro was more efficient at transducing motor neurons compared to other serotypes. Moreover, PHP.S was more efficient at transducing sympathetic neurons, and AAV-retro was more efficient at transducing parasympathetic neurons. These results indicate that specific AAV serotypes target peripheral neuron populations more efficiently than others in the neonatal rat.

## Introduction

Recombinant adeno-associated viruses (AAV) are some of the most widely used viral vectors in basic research because of their long-term efficacy and lack of pathogenicity (Wang et al., 2019). Many different serotypes of AAVs have been either discovered or engineered for different cellular tropisms. Recently there have been improvements in peripheral neuron targeting with AAVs, however, challenges persist due to the anatomical spread of peripheral nerves (Chan et al., 2017; Bedbrook et al., 2018; Chen et al., 2022).

Several AAV vectors have been used to transduce peripheral nerves, including AAV6, AAV8, AAV9, AAV-retro, and AAV-PHP.S (Wang et al., 2005; Yu et al., 2013; Gombash et al., 2014; Jackson et al., 2015; Chan et al., 2017; Chen et al., 2020). AAV9 has been used extensively to transduce cells of the sensory nervous system and has been proven more efficient than other AAV serotypes when delivered systemically in mice (Gombash et al., 2014; Buckinx et al., 2016). AAV9 has also been effective at transducing motor, autonomic, and enteric neurons in mice (Benkhelifa-Ziyyat et al., 2013; Ayers et al., 2015; Wang et al., 2022). PHP.S, engineered from AAV9, has achieved even higher transduction efficacy in both sensory and autonomic nervous systems compared to AAV9 in adult C57Bl/6J mice (Chan et al., 2017; Asencor et al., 2021). AAV-retro has recently been used to transduce peripheral sensory neurons in mouse nodose ganglia (Zhao et al., 2022). Furthermore, AAV-retro efficiently transduces lower motor neurons in the mouse spinal cord, dorsal root ganglia, and trigeminal ganglia (Chen et al., 2020).

In the central nervous system, efficient delivery can be achieved by stereotaxic injection of viral vectors into the nucleus or brain area of interest by transducing cell bodies or nerve endings. In the peripheral nervous system, the nerve endings are more spread out, making efficient and repeatable retrograde targeting challenging (Hoyng et al., 2015; Bedbrook et al., 2018). Further, surgical access to peripheral neuron cell bodies is often more complex. One way to improve transduction efficiency throughout the peripheral nervous system is to use systemic delivery of viral vectors. In general, systemic delivery decreases the anatomical specificity of transduction but increases the number of neurons transduced (Duan, 2016). This technique, paired with Cre- or promotor-restricted expression, can be an effective way to transduce targeted peripheral neuron populations (Bedbrook et al., 2018; Haery et al., 2019).

Studies involving the evaluation of peripheral nerve transduction by using systemic delivery of viral vectors are limited in rat models. While fewer transgenic tools are available, rats offer distinct advantages with their larger anatomy for physiology experiments and are often preferred in more advanced behavioral experiments (Ellenbroek and Youn, 2016). Thus, in this study, we aimed to evaluate the efficiency of the systemic delivery of AAV vectors to the rat peripheral nervous system. We compare AAV9, PHP.S, and AAV-retro in their ability to transduce autonomic, sensory, and motor peripheral neuron populations. We demonstrate that these vectors offer differing transduction efficacy depending on the peripheral nerve type.

## Materials and methods

### Animals

All experiments were performed using post-natal day 1 (P1) pups from timed pregnant Wistar Rats purchased from Envigo. Both male and female rats from born litters were used. They were housed on a 12-h light-dark cycle with standard food and water *ad libitum*. All procedures were approved by the Institutional Animal Care and Use Committee at the University of Florida in strict accordance with the US National Institute of Health (NIH) Guide for the Care and Use of Laboratory Animals. Rats of either gender from born litters were randomly assigned to different injection groups.

### Viruses and injections

All viruses were purchased from Addgene (Watertown, MA, USA). The viruses used were PHP.S-CAG-tdTomato (59462-PHP.S), AAV9-CAG-tdTomato (59462-AAV9), and AAVretro-CAG-GFP (37825-AAVrg). 2.1 × 10^11^ vg/rat were injected intraperitoneally in a volume of 20 μL, and the needle was left in for at least 5 s to equilibrate the liquid and prevent backflow. We then observed the mice for 10 s to look for backflow. We did not observe significant backflow with our injections. To achieve a high vector load per gram of body weight, we injected neonatal rats P1.

### Tissue preparation

Five weeks after transduction, animals were anesthetized with 1.2 g/kg ketamine-xylazine cocktail and transcardially perfused with 0.01 M phosphate-buffered saline (1× PBS) containing heparin (10,000 units). After perfusing with 1× PBS heparin solution, an incision was made in the abdomen to expose the bladder. A 20 G needle was used to inject saline into the bladder to distend it. The rats were then perfused with freshly prepared 4% paraformaldehyde (PFA). Pelvic ganglia, L5-S1 dorsal root ganglia, bladder, and external urethral sphincter were harvested from each rat. We evaluated the lower urinary tract because it has innervation from sensory, parasympathetic, sympathetic, and somatic motor peripheral neurons. This system allowed us to evaluate all these peripheral nervous system branches. The spinal cord was taken in 3 segments: L1–L3, L4–L6, and sacral. Each tissue sample was post-fixed overnight in 4% PFA. Bladders were opened and cut into 1 × 1 mm squares, then stored in 1× PBS with 0.01% sodium azide at 4°C. The dorsal root ganglion, pelvic ganglion, external urethral sphincter, and spinal cord were then transferred to 30% sucrose in 1× PBS at 4°C. After cryopreservation (∼24 h), tissues were embedded in OCT mounting media and stored at −80°C.

### Immunohistochemistry

The dorsal root ganglion, pelvic ganglion, and external urethral sphincter tissues were cross-sectioned using cryostat onto microscope slides (Fisher Scientific, 12-550-15) at 20 μm, 20 μm and 30 μm, respectively. Slides were stored at −20°C until staining. Spinal cords were cross-sectioned using cryostat at 50 μm into wells of 1× PBS and stained as free-floating sections. Tissue sections were permeabilized and blocked for non-specific antibody binding for 1 h at room temperature in the blocking solution (0.3% Triton X-100 with 5–10% normal goat serum diluted in 1× PBS). Tissue sections were incubated with primary antibodies overnight at 4°C, followed by 3 washes in 1× PBS 5-min each (see Table 1 for details on primary and secondary antibodies). After that, sections were incubated with secondary antibodies for 45 min at room temperature. All antibodies were diluted with blocking solution. Slides were coverslipped using mounting media (VectaShield Vibrance Mounting Media, H-1700-10).

**TABLE 1:** Antibodies used for immunohistochemistry.

Antibody target	Host	Source	Product number	Working dilution
β-tubulin	Rabbit	BioLegend, San Diego, CA, USA	802001	1:1000
Tyrosine hydroxylase (TH)	Chicken	Aves Labs, Davis, CA, USA	TYH	1:1000, 1:500
Neuronal nitric oxide synthase (nNOS)	Rabbit	ThermoFisher, Waltham, MA, USA	61-7000	1:500
Calcitonin gene-related peptide (CGRP)	Mouse	abCam, Cambridge, UK	Ab81887	1:1000
Neurofilament 200 (NF200)	Mouse	Sigma, St. Louis, MO, USA	N0142	1:2000
Green fluorescent protein (GFP)	Chicken	abCam, Cambridge, UK	Ab13970	1:2000
Isolectin-IB4 (IB4)	Africa Shrub legume	ThermoFisher, Waltham, MA, USA	I21411	1:1000
Nissl (Neurotrace)	–	ThermoFisher, Waltham, MA, USA	N21483	1:100
Red fluorescent protein	Rabbit	Rockland, Philadelphia, PA, USA	600-401-379	1:1000
Anti-chicken AF488	Goat	Aves Labs, Davis, CA, USA	F-1005	1:1000
Anti-mouse AF488	Goat	ThermoFisher, Waltham, MA, USA	A11001	1:1000
Anti-mouse AF555	Goat	ThermoFisher, Waltham, MA, USA	A21422	1:1000
Anti-chicken AF555	Goat	ThermoFisher, Waltham, MA, USA	A21437	1:1000
Anti-rabbit AF405	Goat	ThermoFisher, Waltham, MA, USA	A31556	1:1000
Anti-rabbit AF647	Goat	ThermoFisher, Waltham, MA, USA	A21244	1:1000

For whole-mount bladder immunohistochemistry, floating sections were stained in a 12-well plate utilizing the same blocking solution described above. Tissues were incubated with primary antibodies for 5–6 days at 4°C on an orbital rocker, followed by washes in 1× PBS and secondary antibodies incubation for 2 h at room temperature on an orbital rocker. Bladder tissues were floated onto glass slides using a paintbrush. Slides were coverslipped with the mounting media mentioned above.

### Image processing and cell quantification

All images were captured and processed using a Keyence BZ-X Series all-in-one fluorescence microscope. Exposure times when capturing images remained constant across images being directly compared. Six images of each animal were taken of different tissue sections for cell quantification in the dorsal root and pelvic ganglion. An experimenter performed all counts using the ImageJ Plugin Colocalization Object Counter (Lunde and Glover, 2020). This plugin allows the user to identify a threshold at which cells can be considered positively labeled in each channel, thus allowing us to determine if a cell is colocalized with viral transduction and a neuronal marker. For antibody staining of neuronal markers, we calculated the lowest noise tolerance that would not identify cells in our control images from the same animals as our experimental group but with no primary antibody. This threshold was determined for each channel that had antibody staining. Any cells with an intensity above this threshold in our experimental images were counted as positive using the colocalization object counter.

For virally transduced channels (Green florescent protein (GFP) or tdTomato), we drew a region of interest (ROI) around 3 experimenter identified non-labeled cells for each animal and then found the lowest noise tolerance (a variable in the Colocalization plugin) that would not identify cells. We calculated the average of these noise tolerances across the 3 ROIs from each animal. Then we calculated a threshold (the average plus two standard deviations) to identify positively transduced cells. Cells above this threshold were considered positive by the semi-automatic counter, and each image was manually checked. Different thresholds were determined for each batch of antibody staining to account for variability between staining groups.

## Data analysis

Data analysis and graphical construction were performed using GraphPad Prism 9 software. Quantitative results are represented as mean ± SEM. Levene’s test for homogenous variances and Shapiro–Wilk test for normalcy was run on all data sets. One-way ANOVA was performed to compare transduction rates between the three viruses. An unpaired student’s *t*-test was performed to compare the percent of transduced neurons that colocalized with each cell marker as well as the percent of each cell marker that colocalized with transduced neurons. For data sets that were normally distributed but had unequal variances, Welch’s *t*-test or Welch’s ANOVA was performed. For data sets that failed the normalcy tests, the Mann–Whitney test was performed. *P*-values of < 0.05 were considered significant (* < 0.05, ^**^ < 0.005, ^***^ < 0.0005, ^****^ < 0.0001 ns = not significant). Each data point on the graphs represents a different animal.

## Results

### Quantification of viral transduction in the sensory and autonomic ganglia after neonatal systemic delivery

**FIGURE 1:**
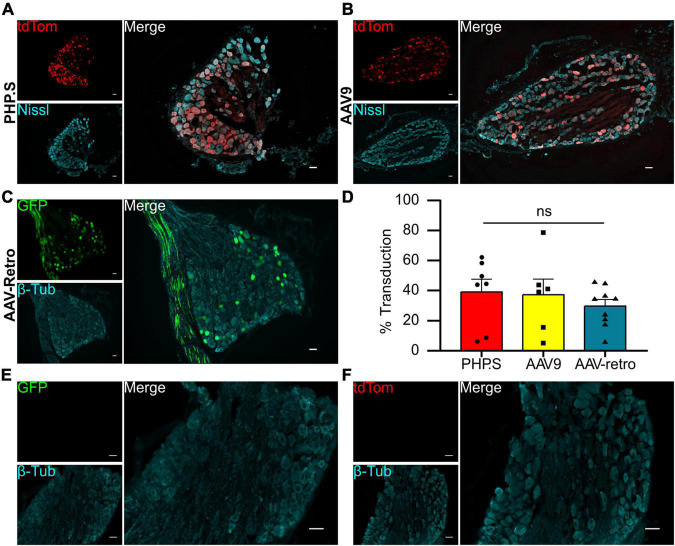
Systemic PHP.S, AAV9, and adeno-associated viruses (AAV)-retro transduce dorsal root ganglion neurons at similar rates. **(A)** Representative image of tdTomato expression in dorsal root ganglion of PHP.S-injected rats. Neurons marked with Nissl (scale bar, 50 μm). **(B)** Representative image of tdTomato expression in dorsal root ganglion of AAV9-injected rats. Neurons marked with Nissl (scale bar, 50 μm). **(C)** Representative image of Green florescent protein (GFP) expression in dorsal root ganglion of AAV-retro-injected rats. Neurons marked with β-tubulin (scale bar, 50 μm). **(D)** Quantification of transduced neurons in dorsal root ganglia using Welch’s ANOVA indicated no significant difference between PHP.S (*n* = 11), AAV9 (*n* = 8), and AAV-retro (*n* = 9) (*p* = 0.4196). **(E,F)** Images of dorsal root ganglia from vehicle-injected rats with the corresponding fluorescent channels for GFP **(E)** and tdTomato **(F)** (scale bar, 50 μm).

**FIGURE 2:**
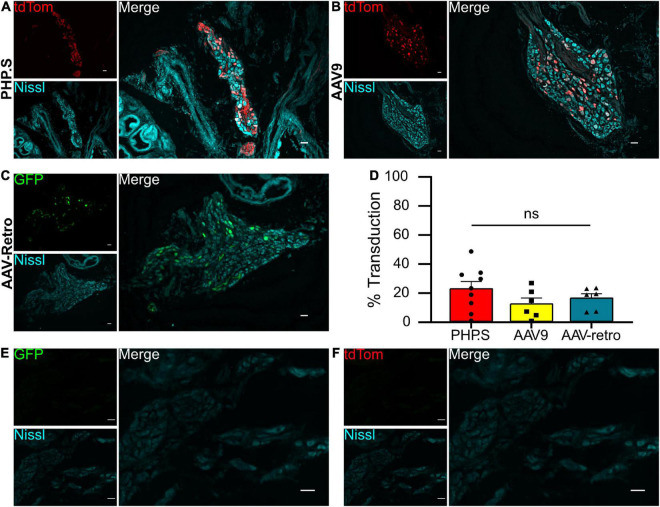
Systemic PHP.S, AAV9, and adeno-associated viruses (AAV)-retro transduce pelvic ganglion neurons at similar rates. **(A)** Representative image of tdTomato expression in the pelvic ganglion of PHP.S-injected rats. Neurons marked with Nissl (scale bar, 50 μm). **(B)** Representative image of tdTomato expression in the pelvic ganglion of AAV9-injected rats. Neurons were marked with Nissl (scale bar, 50 μm). **(C)** Representative image of GFP expression in the pelvic ganglion of AAV-retro-injected rats. Neurons marked with Nissl (scale bar, 50 μm). **(D)** Quantification of transduced neurons in pelvic ganglia using one-way ANOVA indicated no significant difference between PHP.S (*n* = 9 animals), AAV9 (*n* = 6), and AAV-retro (*n* = 6) (*p* = 0.3176). **(E,F)** Images of pelvic ganglia from vehicle-injected rats with the corresponding fluorescent channels for GFP **(E)** and tdTomato **(F)** (scale bar, 50 μm).

We evaluated the viral transduction of systemically delivered viral vectors in sensory and autonomic ganglia after intraperitoneal injections of PHP.S-tdTomato, AAV9-tdTomato, or AAV-retro-GFP post-natal day 1 (P1). Dorsal root ganglia (sensory neurons) and pelvic ganglia (autonomic neurons) were stained with a pan-neuronal marker (Nissl or β-tubulin), and we evaluated the number of neuronal cells transduced with the fluorescent reporter (Figures 1, 2). The transduction efficiency of each virus was quantified by counting the number of Nissl + or β-tubulin + cells that co-expressed tdTomato or GFP in dorsal root ganglion and pelvic ganglion tissue sections (Figures 1D, 2D).

We found that all three viruses exhibited similar levels of neuronal transduction in L5-S1 dorsal root ganglion (PHP.S, 32.9 ± 5.4%; AAV9, 25.4 ± 5.5%; AAV-retro, 25.1 ± 1.5%; ns), whereas dorsal root ganglion from vehicle-injected rats did not show expression in either fluorophore channel (Figures 1D–F). Transduction rates were lower in the pelvic ganglion, with PHP.S transducing the most neurons (Figure 2). However, similar to the dorsal root ganglia, there was no statistical difference between PHP.S, AAV9, and AAV-retro in the transduction of pelvic ganglia neurons (PHP.S, 22.4 ± 5.0%; AAV9, 12.0 ± 4.1%; AAV-retro, 15.9 ± 3.1%; ns) (Figure 2D). Pelvic ganglia taken from vehicle-injected rats did not express any signal in either fluorophore (Figures 2E, F).

**FIGURE 3:**
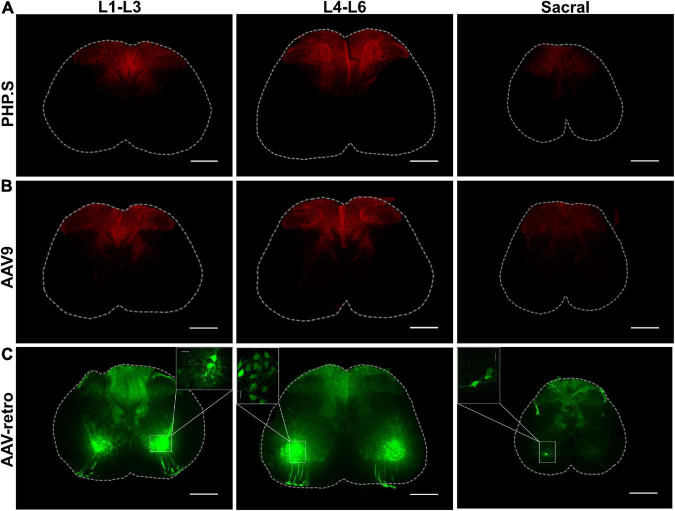
PHP.S, AAV9, and adeno-associated viruses (AAV)-retro transduce sensory neurons innervating the dorsal horn of the spinal cord, while only AAV-retro transduced neurons in the ventral horn. **(A)** Representative images of tdTomato expression from three different spinal cord segments in PHP.S injected animals (scale bar, 500 μm). **(B)** Representative images of tdTomato expression from three different spinal cord segments in AAV9 injected animals (scale bar, 500 μm). **(C)** Representative images of GFP expression from three different spinal cord segments in AAV-retro injected animals (scale bar, 500 μm). The insets magnify cell body transduction in the ventral horn at a lower exposure (scale bar, 50 μm).

We also investigated the different segments of the spinal cord to evaluate peripheral neuron transduction patterns of the neurons innervating the spinal cord. PHP.S and AAV9 groups exhibited similar transduction patterns, centralized to the dorsal horn, consistent with the sensory nerve fibers that innervate the spinal cord from dorsal root ganglion (Figures 3A, B). While AAV-retro demonstrated similar expression as PHP.S and AAV9 in the dorsal horn, it also displayed robust GFP expression in the ventral horn and projections from these regions, which is consistent with motor neuron transduction (Figure 3C).

### Transduction at end organ targets

**FIGURE 4:**
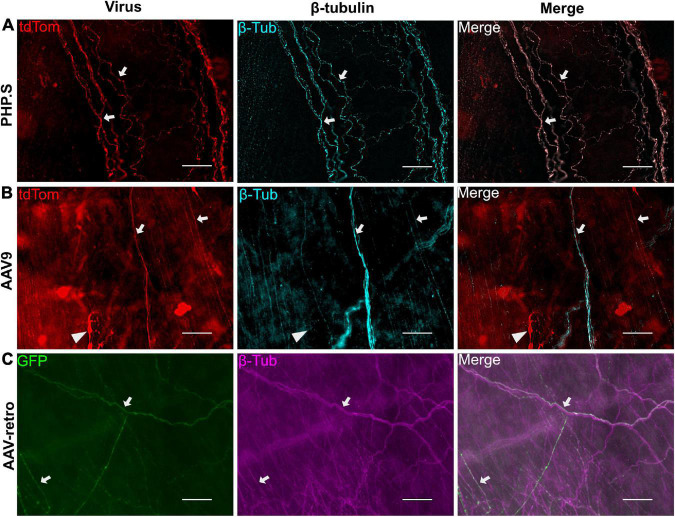
Example of urinary bladder neuronal transduction. **(A)** Representative images from the PHP.S group showing colocalization of β-tubulin with tdTomato (scale bar, 50 μm). **(B)** Representative images from the AAV9 group showing colocalization of β-tubulin with tdTomato (scale bar, 50 μm). The white triangle points to a non-neuronal cell expressing tdTomato. **(C)** Representative images from the AAV-retro group showing colocalization of β-tubulin with GFP (scale bar, 50 μm). White arrows indicate transduced nerve fibers.

**FIGURE 5:**
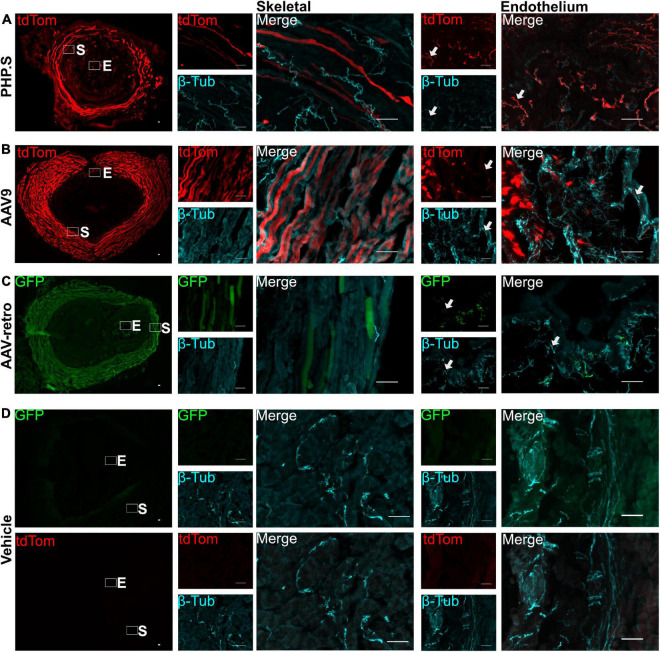
Nerve fibers and non-neuronal cells are transduced by PHP.S, AAV9, and adeno-associated viruses (AAV)-retro in the external urethral sphincter. Representative images of tdTomato **(A,B)** or GFP **(C)** expression in the external urethral sphincter (EUS) (scale bar, 500 μm). Skeletal (S) and endothelium (E) squares are magnified to display expression in the skeletal muscle layers and nerve fibers in the endothelium (scale bar, 50 μm). **(D)** Images of EUS from vehicle-injected rats showing the corresponding fluorophore channels for GFP and tdTomato as well as skeletal muscle and endothelial magnifications (scale bar, 50 μm). White arrows indicate transduced nerve fibers.

We also evaluated the transduction patterns at two end organ targets—the urinary bladder and the external urethral sphincter—that are innervated by the pelvic ganglion, L5-S1 dorsal root ganglion, and motor neurons from lower lumbar and sacral sections. PHP.S, AAV9, and AAV-retro transduced nerve fibers in the bladder were revealed by colocalization with β-tubulin. It was clear that there were fibers that colocalized with β-tubulin and that not all β-tubulin + fibers were virally transduced (Figure 4). Non-neuronal transduction was evident in the external urethral sphincter, where viral expression from all groups was found in the skeletal muscle layers (Figures 5A–C). Immunohistochemistry protocols were performed to amplify tdTomato and GFP in a different channel to confirm that expression was not an artifact (Figures 5A–C). Both PHP.S and AAV-retro groups had expression within the endothelial layer of the external urethral sphincter that colocalized with β-tubulin, confirming nerve fiber transduction lining the lumen, compared to vehicle-injected controls, which did not express any signal (Figure 5D). We also observed some non-neuronal transduction in the bladder (Figure 4B; white arrow).

In the pelvic ganglion, we classified transduced cells as either sympathetic neurons that co-stained with tyrosine hydroxylase (TH) or parasympathetic neurons that co-stained with neuronal nitric oxide synthase (nNOS) (Figures 9, 10). AAV-retro showed significantly higher tropism for sympathetic neurons compared to PHP.S as quantified by the percentage of GFP + or tdTomato + neurons that expressed TH (34.8 ± 3.7 vs. 4.9 ± 3.1%, ^***^p = 0.0002) (Figure 9C). However, there was no statistical difference in the number TH + neurons transduced by AAV-retro compared to PHP.S (22.3 ± 3.6 vs. 8.8 ± 5.1%, *p* = 0.0536) (Figure 9D). Consequently, AAV-retro transduced very few parasympathetic neurons; only 4.2 ± 1.3% of GFP + neurons expressed nNOS. In PHP.S injected animals, 26.6 ± 7.5% of tdTomato + neurons expressed nNOS. PHP.S showed a significant increase in transduced parasympathetic neurons compared to AAV-retro (*p = 0.0394) (Figure 10C). We also saw a significant difference between the percentage of nNOS + neurons transduced by PHP.S compared to AAV-retro (21.0 ± 3.6 vs. 0.7 ± 0.4%, ^**^p = 0.0043) (Figure 10D). We compared antibody labeling to pelvic ganglia with no primary antibody staining (Figures 9E, 10E).

### Classification of transduced dorsal root ganglion neurons

**FIGURE 6:**
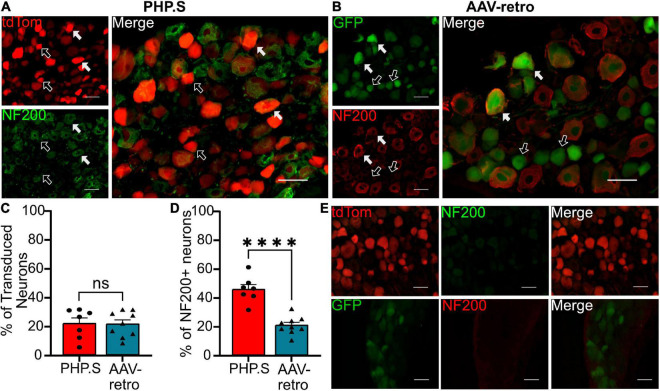
PHP.S and adeno-associated viruses (AAV)-retro transduce large, myelinated neurons at similar rates. **(A)** Representative images of tdTomato expression colocalizing with NF200 + neurons in dorsal root ganglia from PHP.S-injected rats (scale bar, 50 μm). **(B)** Representative images of GFP expression colocalizing with NF200 + neurons in dorsal root ganglia from AAV-retro-injected rats (scale bar, 50 μm). **(C)** Percent of transduced neurons that were NF200 + (*p* = 0.9373). **(D)** Percent of NF200 + neurons transduced by virus (^*⁣*⁣**^p < 0.0001). **(E)** Images of dorsal root ganglia from rats injected with PHP.S (top) or AAV-retro (bottom) stained with secondary antibody only for the corresponding fluorophore used for NF200 in experimental tissue. Statistics were performed using the student’s *t*-test to compare PHP.S (*n* = 7) to AAV-retro (*n* = 9). White arrows indicate neurons with colocalization; black arrows with a white outline indicate viral transduced without colocalization.

**FIGURE 7:**
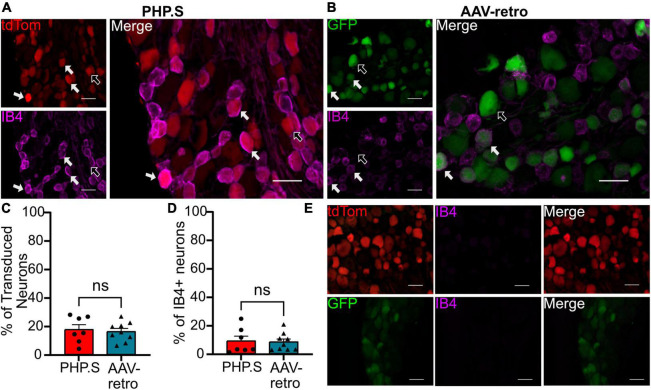
PHP.S and adeno-associated viruses (AAV)-retro transduce small, non-peptidergic neurons at similar rates. **(A)** Representative images of tdTomato expression colocalizing with IB4 + neurons in dorsal root ganglia from PHP.S-injected rats (scale bar, 50 μm). **(B)** Representative images of GFP expression colocalizing with IB4 + neurons in dorsal root ganglia from AAV-retro-injected rats (scale bar, 50 μm). **(C)** Percent of transduced neurons that were IB4 + (*p* = 0.7330). **(D)** Percent of IB4 + neurons that were transduced (*p* = 0.8585). **(E)** Images of dorsal root ganglia tissue from rats injected with PHP.S (top) or AAV-retro (bottom) with no antibody staining in the corresponding fluorophore used for IB4 in experimental tissue. Statistics were performed using the student’s *t*-test to compare PHP.S (*n* = 7) to AAV-retro (*n* = 9). White arrows indicate neurons with colocalization.

**FIGURE 8:**
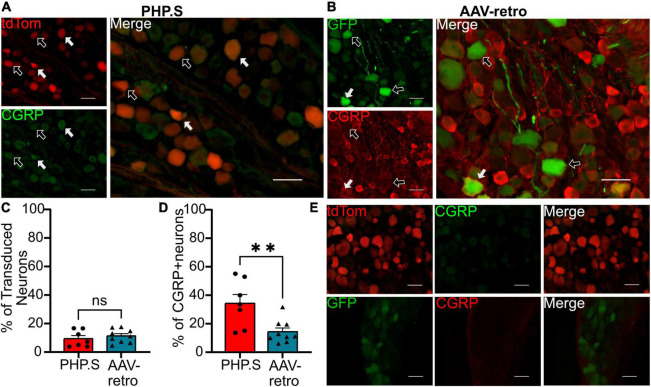
PHP.S and adeno-associated viruses (AAV)-retro transduces small, peptidergic neurons at similar rates. **(A)** Representative images of tdTomato expression colocalizing with calcitonin gene-related peptide (CGRP) + neurons in dorsal root ganglia from PHP.S-injected rats (scale bar, 50 μm). **(B)** Representative images of GFP expression colocalizing with CGRP + neurons in dorsal root ganglia from AAV-retro-injected rats (scale bar, 50 μm). **(C)** Percent of transduced neurons that were CGRP + (*p* = 0.4728). **(D)** Percent of CGRP + neurons that were transduced (^**^p = 0.0066). **(E)** Images of dorsal root ganglia from rats injected with PHP.S (top) or AAV-retro (bottom) stained with secondary antibody only for the corresponding fluorophore used for CGRP in experimental tissue. Statistics were performed using the student’s *t*-test to compare PHP.S (*n* = 7) and AAV-retro (*n* = 9). White arrows indicate neurons with colocalization; black arrows with a white outline indicate viral transduced without colocalization.

We next sought to classify what types of sensory and autonomic neurons were transduced by PHP.S and AAV-retro. Due to the similarity of broad transduction of AAV9 and PHP.S, we chose only to compare PHP.S and AAV-retro. We used three markers to classify different neuronal populations: neurofilament 200 (NF200) for large, myelinated neurons, calcitonin gene-related peptide (CGRP) for small, non-myelinated, peptidergic neurons, and isolectin-B4 (IB4) for small, non-myelinated, non-peptidergic neurons (Figures 6–8). Both PHP.S and AAV-retro showed similar tropism for NF200 + neurons, as quantified by the percentage of tdTomato + or GFP + neurons that expressed NF200 (21.5 ± 3.9 vs. 21.1 ± 2.9%, ns) (Figure 6C). We also saw no significant difference in the number of NF200 expressing neurons transduced by PHP.S or AAV-retro. The percentage of NF200-positive neurons transduced by the virus was significantly higher in the PHP.S group compared to the AAV-retro group (45.1 ± 3.4 vs. 20.5 ± 2.0%, ^****^p < 0.0001) (Figure 6D). Alternatively, both viruses showed a lower affinity for IB4 + neurons with no significant difference in percent tdTomato + expressing IB4 and percent GFP + expressing IB4 (17.2 ± 3.5 vs. 15.8 ± 2.2%, ns) (Figure 7C). We found no significant difference between the percent of IB4 + neurons expressing PHP.S vs. AAV-retro (8.8 ± 3.4 vs. 8.1 ± 2.1%, ns) (Figure 7D). There was no significant difference between tdTomato + neurons expressing CGRP and GFP + neurons expressing CGRP (9.2 ± 2.2 vs. 11.1 ± 1.5%, ns) (Figure 8C). PHP.S transduced CGRP-expressing neurons in the dorsal root ganglia at a significantly higher rate than by AAV-retro (33.8 ± 6.2 vs. 14.1 ± 2.7%, ^**^p = 0.0066) (Figure 8D). Dorsal root ganglia with no primary antibody were used to compare labeled to unlabeled cells (Figures 6E, 7E, 8E).

**FIGURE 9:**
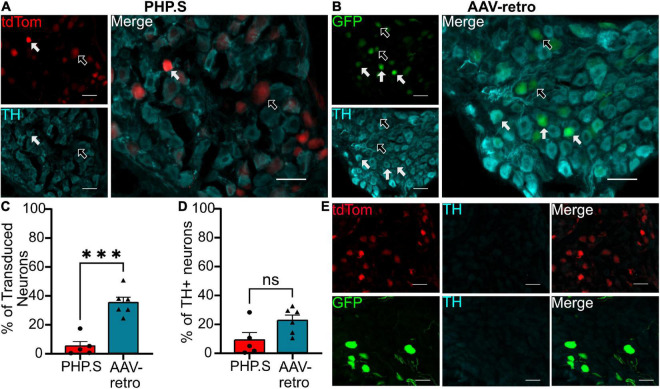
Adeno-associated viruses (AAV)-retro shows tropism for sympathetic neurons over PHP.S in pelvic ganglia. **(A)** Representative images of tdTomato expression colocalizing with TH + neurons in pelvic ganglia from PHP.S-injected rats (scale bar, 50 μm). **(B)** Representative images of GFP expression colocalizing with TH + neurons in pelvic ganglia from AAV-retro-injected rats (scale bar, 50 μm). **(C)** Percent of transduced neurons that were TH + (^***^p = 0.0002). **(D)** Percent of TH + neurons that were transduced (*p* = 0.0536). **(E)** Images of pelvic ganglia from rats injected with PHP.S (top) or AAV-retro (bottom) with secondary antibody staining only in the corresponding fluorophore used for TH in experimental tissue. Statistics were performed using the student’s *t*-test to compare PHP.S (*n* = 5) and AAV-retro (*n* = 6). White arrows indicate neurons with colocalization; black arrow with a white outline indicates viral transduced without colocalization.

**FIGURE 10:**
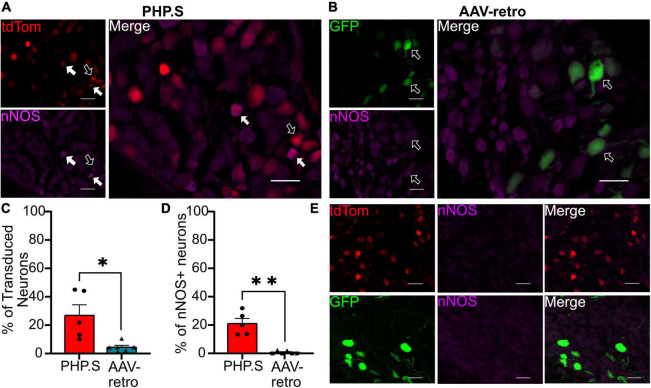
PHP.S shows tropism for parasympathetic neurons over adeno-associated viruses (AAV)-retro in pelvic ganglia. **(A)** Representative images of tdTomato expression colocalizing with neuronal nitric oxide synthase (nNOS) + neurons in pelvic ganglia from PHP.S-injected rats (scale bar, 50 μm). **(B)** Representative images of GFP expression colocalizing with nNOS + neurons in pelvic ganglia from AAV-retro-injected rats (scale bar, 50 μm). **(C)** The percent of transduced neurons that were nNOS +, Welch’s *t*-test was used to compare PHP.S (*n* = 5) and AAV-retro (*n* = 6) because the data set had unequal variances (*p = 0.0394). **(D)** Percent of nNOS + neurons that were transduced. Mann–Whitney test was used to compare PHP.S (*n* = 5) and AAV-retro (*n* = 6) because the data had unequal variances and did not reach normalcy (*p = 0.0043 and ^**^p = 0.0043). **(E)** Images of pelvic ganglia tissue from rats injected with PHP.S (top) or AAV-retro (bottom) with secondary antibody staining only in the corresponding fluorophore used for nNOS in experimental tissue. White arrows indicate neurons with colocalization; black arrow with a white outline indicates viral transduced without colocalization.

## Discussion

AAVs are widely used in basic science research for targeted genetic manipulation of specific neuronal populations. While there is extensive work evaluating the use of different AAV serotypes in the central nervous system (Foust et al., 2009; Jackson et al., 2015; Lukashchuk et al., 2016; Rincon et al., 2018), there are fewer studies assessing transduction of the peripheral nervous system (Wang et al., 2005; Zincarelli et al., 2008; Gombash et al., 2014; Buckinx et al., 2016) and even fewer in rats (Yu et al., 2013). We aimed to determine the transduction abilities of three AAV vectors in the rat peripheral nervous system: PHP.S, AAV9, and AAV-retro. We did not observe an appreciable difference between the three viruses regarding the transduction efficiency in sensory or autonomic neuronal populations. However, there was a significant difference between the transduction of the parasympathetic and sympathetic autonomic branches. AAV-retro transduced significantly more sympathetic neurons than PHP.S and significantly fewer parasympathetic neurons than PHP.S. Additionally, we found that AAV-retro was transducing spinal cord neurons in the ventral horn where motor neurons are located, which we did not see in the PHP.S or AAV9 groups.

AAV-PHP.S was engineered from the AAV9 serotype to have higher transduction efficiency in the peripheral nervous system in adult mice (Chan et al., 2017). We investigated if this pattern would be consistent in rats. Our data revealed no significant difference between the engineered PHP.S and AAV9 when delivered systemically in neonatal rats. The discrepancy between mice and rats could be due to a species difference or related to the dose of viral particles administered. The original study comparing PHP.S to AAV9 used a much higher dose of virus (1 × 10^12^ vg/mouse) than we did (2.1 × 10^11^ vg/rat), so the increased efficiency of PHP.S may only occur at higher concentrations (Chan et al., 2017). Furthermore, we delivered the viruses in neonates, while Chan et al. delivered the viruses in adult animals. Developmental differences in transduction between P0 and P2 administration have been reported (Chakrabarty et al., 2013), while other studies have observed better transduction in neonates than in adults (Zheng et al., 2010), indicating that the developmental cycle may affect transduction efficiency.

Both PHP.S and AAV9 exhibit broad transduction of the peripheral nervous system and are viable options for rat model studies, but PHP.S does not seem to offer an appreciable increase in transduction compared to AAV9, despite being engineered for optimized performance. The same group that developed PHP.S recently published two new vectors: AAV-MaCPNS1 and AAV-MaCPNS2 (Chen et al., 2022). These vectors have optimized peripheral transduction across multiple species and have better transduction in adult animals compared to AAV9 and PHP.S. Future work will need to evaluate the systemic delivery of these vectors to neonatal rats.

Retrograde tracers such as AAV-retro enter the cell through axon terminals and travel to the cell body, labeling cells at the projection site (Xu et al., 2020). We evaluated AAV-retro delivered systemically in rats as it had been shown to broadly transduce motor neurons when given intramuscularly in mice (Chen et al., 2020). Although Chen et al. did not quantify expression in sensory neurons, they did observe extensive transduction of dorsal root ganglion neurons. This finding is similar to our results, which show high transduction in the dorsal root ganglion of neonatal rats injected intraperitoneal.

We also found comparable transduction patterns in the spinal cord of AAV-retro injected rats as Chen et al. found in mice injected with AAV-retro intramuscular. AAV-retro exhibited robust transduction of spinal cord motor neurons, notably in the ventral horn, compared to other viral vectors, indicating that it has potential for studies targeting lower motor neurons. However, because Chen et al. delivered the virus into the forelimb on one side, they observed reduced transduction in the contralateral spinal cord, whereas we found relatively equal transduction on both sides.

We observed similar transduction patterns across the three groups in the dorsal horn, consistent with data collected from the transduction in the dorsal root ganglia. Furthermore, Chen et al. reported significantly higher spinal motor neuron transduction levels with AAV-retro than with AAV1, AAV2, and AAV5-AAV9 after intramuscular delivery in neonatal mice. Our data support these findings as AAV-retro also transduced neurons in the ventral horn; ventral horn transduction was absent with AAV9 or PHP.S. In contrast, other studies showed robust transduction of lower motor neurons in the spinal cord with AAV9 *via* systemic delivery to neonatal mice and rats (Foust et al., 2009; Jackson et al., 2015). Aside from species differences, the discrepancy could be explained by the amount of vector delivered to each animal. Jackson et al. found that an increased dose of AAV9 vector genomes led to higher transduction efficiency in adult mice than the lower dose initially used. This means that AAV9, AAV-retro, and possibly PHP.S have the potential to transduce lower motor neurons, but AAV-retro may be the preferred vector because it requires a lower dose.

PHP.S, AAV9, and AAV-retro transduced nerve fibers in the bladder and external urethral sphincter. PHP.S and AAV-retro transduced nerve fibers in the endothelial layer of the urethra, which are likely sensory fibers. All three viruses also transduced nerve fibers in the skeletal muscle of the external urethral sphincter. In the bladder, we found some non-neuronal transduction of an unconfirmed cell type. Future studies can focus on classifying the types of nerve fibers and cells transduced by these viruses in the external urethral sphincter and bladder. These results indicate that we may see off-target transduction when using systemic delivery, and this would need to be controlled for and considered when interpreting results.

Promoters offer a way to select which cell types are being transduced. Because AAVs have such a small capsid, AAV-compatible promoters typically abbreviated to fit these viral vectors’ packing capacity could be used (Gray et al., 2011; de Leeuw et al., 2014). However, this comes with the balance of expression level, as using a cell type-specific promoter may diminish this robust expression depending on the targeting promotor. Here we used the universal short CMV early enhancer/chicken β-actin (CAG) promoter to drive robust expression in all cell types.

Additionally, it is possible to place complementary microRNA binding sites into the vector genome to repress off-target transduction (Xie et al., 2011). Using Cre recombinase in tandem with AAVs has also provided another method of targeted gene transfer and a way to deliver Cre to adult animals (Ahmed et al., 2004). Cre-recombination-based AAV targeted evolution has led to engineered AAV capsids with higher tropism than the serotype they originated from, such as PHP.S, AAV-MaCPNS1, and AAV-MaCPNS2 from AAV9 in mice (Deverman et al., 2016; Chan et al., 2017; Chen et al., 2022). Other viruses, such as lentivirus and HSV, have also been used for gene transfer studies because of their larger carrying capacity compared to AAVs and thus the ability to integrate large promotor sequences to gain specificity (Yu et al., 2011; Shimizu et al., 2017).

Although the three viruses showed similar levels of transduction in the dorsal root ganglion, our immunohistochemistry classification studies revealed levels of tropism for different neuronal cell types (Figures 6–8). Although PHP.S and AAV-retro did not exhibit a significant difference in tropism for dorsal root ganglia neurons, they both transduced large, myelinated neurons more than small, non-myelinated neurons (Figures 6–8). This data is supported by Skorput et al. (2022), who evaluated the mean size of somas being transduced by AAV-retro in dorsal root ganglia and saw that AAV-retro was transducing large-soma neurons. AAV9, which PHP.S was derived from, also transduces medium-to-large dorsal root ganglion neurons (Kubota et al., 2019).

There was no difference in the percentage of transduced neurons that were either CGRP + or NF200 + in PHP.S and AAV-retro groups. However, we did observe a significant increase in the percent of CGRP + and NF200 + neurons transduced by PHP.S compared to AAV-retro (Figures 6, 8). In other words, PHP.S transduced a greater population of the total CGRP + and NF200 + dorsal root ganglion cells. So while AAV-retro and PHP.S capsids broadly transduced the same proportions of CGRP + and NF200 + cells (∼10% of transduced neurons were CRGP + and ∼20% of transduced neurons are NF200 +), PHP.s seems to transduce more of the CGRP (33.8 ± 6.2 vs. 14.1 ± 2.7%) and NF200 (45.1 ± 3.4 vs. 20.5 ± 2.0%) population. There was no statistical difference in overall transduction efficacy in the dorsal root ganglion comparing AAV9, PHP.S, and AAV-retro; PHP.S had the highest efficiency at ∼32% compared to AAV-retro at ∼25% (Figure 1). Altogether, these results indicate that PHP.s may have a better transduction efficiency in CGRP + and NF200 + cell populations.

We looked at viral transduction in the pelvic ganglion, which contains sympathetic and parasympathetic neurons innervating the lower urinary tract and other pelvic organs. We found that AAV-retro preferentially transduces sympathetic neurons and almost no parasympathetic neurons. On the other hand, PHP.S transduced parasympathetic neurons more than sympathetic neurons and at a significantly higher rate than AAV-retro. These transduction patterns can be considered valuable tools in focused genetic manipulation of these autonomic neuron populations.

In terms of the limitations of this study, it is important to note that we were working with relatively small sample sizes. Due to the limited availability of animals, we could only sample five to seven animals for each group. This limited our ability to compare sex differences, which will be necessary for understanding the transduction profiles of the viral vectors. Future research should compare sex differences as well as different routes of systemic administration. Further, we did see variability within our groups. There are several potential reasons for this, including how much of the virus got systemically circulated from the intraperitoneal injection, whether some virus leaked out of the injection site following the injection observation period and potential variability in neonatal immune responses. Considering these variables and the variability we observed when designing future experiments using this technique is essential.

In summary, these AAV viruses can transduce peripheral neurons in rats. Although PHP.S was optimized from the AAV9 serotype for sensory neuron transduction in mice, our results indicate no advantage to using PHP.S over AAV9 in rats. Between AAV-retro, PHP.S, and AAV9, no one virus exhibits better transduction efficiency of sensory or autonomic populations. However, AAV-retro may have an edge when transducing motor neurons and neurons of the sympathetic nervous system. Additionally, PHP.S has an advantage when transducing parasympathetic neurons over AAV-retro.

## Data availability statement

The raw data supporting the conclusions of this article will be made available by the authors, without undue reservation.

## Ethics statement

The animal study was reviewed and approved by Institutional Animal Care and Use Committee at the University of Florida.

## Author contributions

OY and AM contributed to the conception, design of the study, and wrote sections of the manuscript. OY, GR, FA, KA, KD, and AM contributed to collecting and analyzing the data, histology, microscopy, and animal injections and care. OY assembled and designed the figures. AM supervised and acquired funding for this project. All authors contributed to manuscript revision and read and approved the submitted version.
